# Health-related quality of life in Norwegian adolescents living with chronic fatigue syndrome

**DOI:** 10.1186/s12955-020-01430-z

**Published:** 2020-06-05

**Authors:** Wenche Ann Similä, Vidar Halsteinli, Ingrid B. Helland, Christer Suvatne, Hanna Elmi, Torstein Baade Rø

**Affiliations:** 1 Children’s Clinic, St. Olavs Hospital, Trondheim, Norway; 2 Department of Clinical and Molecular Medicine, Norwegian University of Science and Technology, N-7489 Trondheim, Norway; 3 Regional Center for Health Care Improvement (RSHU), St. Olavs Hospital, Trondheim, Norway; 4 Department of Public Health and Nursing, Norwegian University of Science and Technology, Trondheim, Norway; 5 Division of Pediatric and Adolescent Medicine, Oslo University Hospital, Oslo, Norway

**Keywords:** Adolescent, Adolescent health, Chronic fatigue syndrome, Quality of life

## Abstract

**Purpose:**

The primary aim was to measure health related quality of life (HRQoL) in a Norwegian cohort of adolescents with Chronic Fatigue Syndrome (CFS/ME). A secondary aim was to identify factors before diagnosis, at time of diagnosis and after diagnosis that were associated with HRQoL.

**Methods:**

In this cross-sectional population-based study, HRQoL was measured by Pediatric Quality of Life Inventory™ Generic Core scale version 4.0 (PedsQL4.0) in 63 adolescents with CFS/ME. In addition, fatigue was measured by PedsQL Multidimensional Fatigue scale (PedsQL-MFS), depressive symptoms were measured by the Short Mood and Feelings Questionnaire (SMFQ), and disruption in school activities was measured by The De Paul Pediatric Health Questionnaire (DPHQ-N). Data were also collected from medical records and patient interviews.

**Results:**

Age at diagnosis was 15 (2) years (mean (SD)), and four out of five participants were female. Time from diagnosis to reply was 39 (22) months. Adolescents with CFS/ME reported PedsQL4.0 score 50 (17), and boys reported a better score than girls (64 vs 47, 95% Confidence Interval (CI) for difference (− 27; − 6)). There were positive associations between overall HRQoL and support from a schoolteacher, school attendance or participation in leisure activities. There were negative associations between overall HRQoL and delayed school progression, having been to rehabilitation stay and depressive symptoms.

**Conclusion:**

HRQoL in adolescents diagnosed with CFS/ME was low. The associations between reported HRQoL, healthcare previously provided, support from a schoolteacher, school attendance and participation in leisure activity may provide information of value when developing refined strategies for healthcare among adolescents with CFS/ME. Possible causal relationships must however be explored in future studies.

## Introduction

Chronic fatigue syndrome (CFS) is characterized by overwhelming and severe disabling fatigue, and loss of physical and mental endurance [1]. The condition is also cited as Myalgic Encephalomyelitis (ME) to conceptualize a specific neuroimmunological condition [2]. A main characteristic of the disease is post exertional malaise (PEM). Other symptoms include orthostatic intolerance and other signs of autonomic dysfunctions, cognitive impairment, unrefreshing sleep, sore throat, headache, dizziness, heat and cold intolerance, muscular and abdominal pain, nausea, vomiting and mood disturbances [3–6]. CFS/ME occurs more frequently in the age groups 11–19 and 30–39 and is 3–4 times more common in girls than boys. In Norway 0,1–1,0% adolescents are affected [1, 7, 8]. Adolescents CFS/ME starts with an acute or gradual, infectious or non - infectious onset. Establishment of the diagnosis is frequently delayed, with the period from start of symptoms to diagnosis varying from 5 to 17 months [3, 9]. The CFS/ME disease course is often measured in years, and it is common to experience recurring improvement and relapses [1]. Among adolescents, CFS/ME is the most common cause of long-term absence from school [4, 10–13].

Previous studies have shown that CFS/ME severely impact health-related quality of life (HRQoL) [1]. Typically, adolescents with CFS/ME report lower HRQoL than adolescents diagnosed with other chronic health conditions like ADHD, cancer or cerebral palsy [14–17]. In an earlier Norwegian study of HRQoL among adolescents with CFS/ME, patients scored 49 whereas healthy controls scored 93 on a 0–100 generic HRQoL scale based on PedsQL 4.0 [16].

CFS/ME patients need care from primary health care and schools. Because of the complexity and severity of the illness, specialized care from personnel experienced with CFS/ME is often warranted, but frequently not available [1]. Despite substantial efforts from health care and schools, we still lack knowledge about effective strategies to improve disease outcome and HRQoL. The main aim of this study was to measure HRQoL in adolescents with CFS/ME, and a secondary aim was to identify factors before diagnosis, at diagnosis and after diagnosis positively or negatively associated with HRQoL.

## Methods

### Study design

A cross-sectional, population-based study of HRQoL in adolescents diagnosed with CFS/ME.

### Study population

#### CFS/ME adolescent patients

Adolescents diagnosed with CFS/ME at St. Olavs or Oslo University Hospitals in Norway with age 12–18 at the time of diagnosis were invited by mail to participate. Participants were asked to complete a questionnaire and to attend an interview. Invitation was sent between August 2017 and January 2018, and time since diagnosis varied from 1 to 118 months. Of 168 invited, 86 (51,2%) agreed to participate, and 63 (37,5%) returned completed questionnaires. All participants were diagnosed with G 93.3 CFS/ME according to Jason diagnostic criteria [5], and the diagnosis was verified by an independent evaluation of medical records. Exclusion criteria were not being able to read Norwegian or reply to questionnaires or participate in interview. No one was excluded according to these criteria. Data collection from questionnaires, interviews and medical records finished in June 2018.

### Measures

#### PedsQL generic Core scale

The Norwegian version of Pediatric Quality of Life Inventory™ Generic Core scale version 4.0 (PedsQL4.0) was used to measure HRQoL. PedsQL4.0 is a 23-item generic questionnaire developed to measure HRQoL in both healthy and acute or chronic ill children and adolescents [18]. A young adult version was used for ages 18–22. The PedsQL4.0 provides a generic sum score and subscale scores; Physical functioning (8 items) and Psychosocial functioning as total of: Emotional functioning (5 items), Social functioning (5 items) and School functioning (5 items). Participants were asked to rate each item during the last month on a Likert scale from 0 (never a problem) to 4 (almost always a problem). The items were reversely scored and linearly transformed on a scale ranging from 0 to 100 (0 = 100, 1 = 75, 2 = 50, 3 = 25, 4 = 0). Higher scores indicate better HRQoL [18].

#### PedsQL multidimensional fatigue scale

The Pediatric Quality of Life Inventory™ Multidimensional Fatigue scale (PedsQL-MFS) was used to measure fatigue severity [19]. PedsQL-MFS is a generic scale with 18 items, and with subscale scores for three domains; general fatigue (6 items), fatigue related to sleep/rest (6 items) and cognitive fatigue (6 items). The response scale is the same as for PedsQL4.0. Higher scores indicate less fatigue.

#### Short mood and feelings questionnaire

The Short Mood and Feelings Questionnaire (SMFQ) is a self-report-form measuring depressive symptoms in children and adolescents. Thirteen items collectively describe depressive symptoms, covering for symptoms specific for major depression in DSM-IV. The SMFQ items have three reply options; “True” =2, “Sometimes” =1, “Not true” =0 referring to the last 2 weeks with a sum score from 0 to 26. A sum score of 11 or higher indicates depressive symptoms which possibly require treatment [20].

#### De Paul pediatric health questionnaire – Norwegian version

The De Paul Pediatric Health Questionnaire (DPHQ-N) for children and adolescents was translated to Norwegian by The Norwegian National Advisory Unit on CFS/ME. Translation and re-translation was performed according to EORTC [21], re-translated by one and accepted by original author. This is a self-report questionnaire for children ages 10–17 in three parts; 1) demographic data, 2) a list of CFS/ME-related symptoms from the current CFS/ME criteria according to Jason, with symptoms rated in frequency (0 = never, 1 = almost never, 2 = half the time, 3 = almost always, 4 = always), and in severity (0 = no problem, 1 = small problem, 2 = moderate problem, 3 = big problem, 4 = very big problem), and 3) experience of disruption in school activities or performance due to fatigue or cognitive difficulties [5, 22, 23].

#### Data from medical records and additional interviews with patients

Data from medical records were collected using a semi-structured guide developed by the research group. One pediatrician from each hospital and one psychiatrist from St. Olavs hospital used the same guide when collecting data from diagnostic evaluation in medical records. Supplementary data providing information of contact with primary health care personnel and schools were collected directly from the participants via a six to 7 minute telephone interview using an interview guide with the same questions for all participants.

### Statistical analyses

First, we examined the study population characteristics. Then we examined HRQoL, fatigue and depressive symptoms in relation to study population characteristics. Continuous data (age, duration of fatigue, HRQoL, fatigue severity and depressive symptom scores) are presented as mean (SD), Median (Q1-Q3), and 95% confidence intervals (CI) where appropriate. Correlations between HRQoL, fatigue severity and depressive symptoms are presented as Pearson’s correlation coefficients. Categorical data (gender, recovered/not recovered, delayed school progression and participation in leisure activities) are presented as numbers and percentages.

Next we examined a wide spectrum of factors related to the periods before diagnostic evaluation, through diagnostic evaluation and after diagnosis in relation to HRQoL (PedsQL 4.0). For these dichotomous factors two-sided independent sample t-test were used to assess differences. Complete results are presented in Supplemental Tables 1, 2, 3, 4, 5, 6, while statistically significant variables (5% level) are presented in Table 3. Generic HRQoL scores are presented as mean (SD) plus 95% confidence intervals (CI) for differences. For dimension scores only difference and CI are reported. These analyses were indicators for regression analysis and were not corrected for multiple testing.

Finally, we examined independent variables significantly associated with HRQoL as predictor variables in a multiple linear regression model, controlling for gender. The model included all participants with replies to dichotomous variables. Dependent variable was tested for normal distribution and outliers. Predictor variables were tested for multicollinearity. Unstandardized Beta coefficients with confidence intervals (CI), Adjusted R^2^, model significance (ANOVA), F-values, β-values and *p*-values for each predictor were reported. A difference of 10 in primary outcome (HRQoL) was predetermined as clinically relevant.

Among 63 participants, 48 answered additional questions about contact with primary health care personnel and school. All statistical analyses were performed using SPSS version 23.

### Ethics

The study was approved by The Regional Ethical Committee for medical and health profession research in Norway (REK 2017/749). All participants signed an informed consent, and the study was performed according to the declaration of Helsinki. The participants were offered a consultation to explore the need for further health care.

## Results

### Study population characteristics

Sixty-three adolescents with CFS/ME were included in the study, with a female: male ratio of 4,2:1 and mean age18 years (Table 1). Duration of fatigue initial to diagnostic evaluation was 15 months (10–33) (median (Q1-Q3)), and at the time of study enrollment 52 months (36–67) (median (Q1-Q3)). 37 (76%) of the adolescents had a delayed school progression defined as not having completed all compulsory subjects in school at their level. Furthermore, most of the adolescents (66%) reported no participation in leisure activities. Four adolescents had recovered after 6, 12 and 36 months, respectively (one unknown) (Table 2).

**Table 1:** Patient characteristics and overall results from PedsQL 4.0, PedsQL-MFS and SMFQ

	N	%	Mean (SD)	Median (Q1-Q3)
Gender (Female / Male / Undetermined)	50/12/1	79/19/2		
Age at time of enrolment	63		18 (2)	
Duration of fatigue before diagnostic evaluation (months) **a)**	48			15 (10–33)
Duration of fatigue at time of enrolment (months)	59			52 (36–67)
Recovered from CFS/ME (yes/no)	4/58	6/94		
Delayed school progression (yes/no)	37/12	76/24		
Participation in leisure activities (yes/no)	21/41	34/66		
**PedsQL4.0**				
Overall HRQoL score **(b)(c)**	63		50 (17)	
Sub-scale scores: Social functioning	63		67 (16)	
Emotional functioning	63		56 (20)	
Physical functioning	63		42 (25)	
School functioning	58		41 (21)	
Psychosocial functioning	63		55 (16)	
**PedsQL-MFS**				
Overall Fatigue score **(b)**	62		36 (19)	
Sub-scale scores: Cognitive fatigue	62		41 (25)	
Fatigue related to sleep/rest	62		36 (19)	
General fatigue	62		32 (23)	
**SMFQ (13 items)** Sum score **(c)**	63		7 (5)	
Score < 11 / 11 or higher	46/17	73/27		

a) Not available data from 15 participants, b) Pearson’s correlation = .861, *p* < .001 between generic PedsQL4.0 and overall PedsQL-MFS, c) Pearson’s correlation = −.544, *p* < .001between generic PedsQL 4.0 and SMFQ sum score

**Table 2:** PedsQL generic and multidimensional fatigue scales and SMFQ sum score related to patient characteristics

	*PedsQL Generic scale (23 items)*			*PedsQL Multidimensional Fatigue scale (18 items)*			*SMFQ sum score (13 items)*		
	***N***	Mean (SD)	95%CI for diff	***N***	Mean (SD)	95%CI for diff	N	Mean (SD)	95%CI for diff
Gender									
Girls	50	47 (16)	*(−27; −6)*	49	33 (16)	*(−30; −7)*	50	8 (5)	*(2–6)*
Boys	12	64 (20)		12	51 (24)		12	4 (3)	
Age:									
< 16	10	57 (17)	*(−4–20)*	10	46 (13)	*(−2; − 24)*	10	6 (3)	*(−4–0)*
16+	53	49 (17)		52	34 (20)		53	8 (6)	
Status at response time									
Not recovered from CFS/ME	58	48 (16)	*(19–51)*	57	33 (16)	*(−61; −28)*	58	8 (5)	*(0–10)*
Recovered from CFS/ME	4	83 (13)		4	78 (15)		4	3 (4)	
Is your school progression delayed									
Yes	37	49 (16)	*(−27; −4)*	37	34 (19)	*(−31; −6)*	37	8 (5)	*(−1–6)*
No	12	65 (18)		12	52 (21)		12	5 (6)	
School attendance before diagnosis									
< 50%	39	50 (18)	*(−25–2)*	38	36 (19)	*(−29–1)*	39	8 (5)	*(− 2–6)*
50% or more	9	61 (21)		9	50 (25)		9	6 (5)	
Do you participate in leisure activity									
Yes	21	59 (17)	*(4–22)*	21	46 (19)	*(5–24)*	21	6 (4)	*(−5–0)*
No	41	46 (16)		40	31 (18)		41	9 (5)	

Two-sided Independent Samples T-test. Difference in N is due to one undetermined which gender, and that participation with questionnaire was higher than participation in interview

Generic PedsQL4.0 score for all participants in this study was 50 (17) (mean (SD)) (Table 1). The subscale scores were lowest for the domain school functioning and highest for social functioning. Overall PedsQL-MFS score was 36 (19). The domain general fatigue had the lowest score. There was a strong correlation between generic PedsQL4.0 and overall PedsQL-MFS scores (Pearson’s *r* = .861, *p* < .001). The SMFQ sum score was 7 (5) (mean (SD)), whereas 27% of the participants scored 11 or higher, suggesting a possible treatment-requiring depression. The correlation between SMFQ and generic PedsQL4.0 score showed moderate to strong negative correlation (*r* = −.544, *p* < .001).

**Fig. 1:**
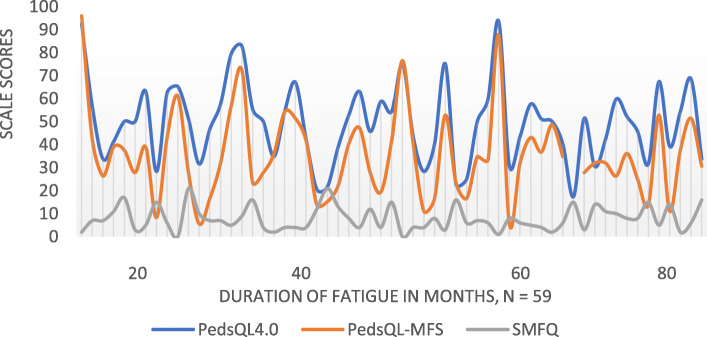
HRQoL-, Fatigue- and SMFQ- scores in relation to Duration of fatigue, *n* = 59. For PedsQL4.0 and PedsQL-MFS high score is better, for SMFQ low score is better

There was no correlation between duration of fatigue at time of study enrolment and overall score of HRQoL, fatigue level or depressive symptoms (Fig. 1). CFS/ME diagnosis for all participants were confirmed from medical records. In DPHQ-N question 59 IV “Worst symptom right now” there was an option to mark “I am not ill” – 4 participants marked this option at the time of reply. This was recorded as recovery from CFS/ME. Adolescents recovered from CFS/ME reported higher HRQoL than those who had not recovered (83 vs. 48, *p* < .001) (Table 2).

### HRQoL, fatigue and depressive symptoms versus study population characteristics

There was a significant gender difference in generic PedsQL4.0 score where girls scored significantly lower than boys (47 vs 64, *p* = .003). In subscale scores, girls scored lower than boys for all dimensions (data not shown). There was also a significant gender difference in PedsQL-MFS score where girls scored lower than boys, and a similar result was found in the SMFQ score (Table 2).

School attendance, delayed school progression or participation in leisure activities were not statistically significant associated with SMFQ scores (Table 2). However, both generic PedsQL4.0 and overall PedsQL-MFS scores differed between adolescents having or not having participated in leisure activities or delayed school progression, with higher scores for the adolescents who were able to participate in leisure activities and adolescents with a normal school progression. School attendance before diagnostic evaluation showed a similar trend when setting the cut-off at 50% school attendance, although not attaining statistical significance.

### HRQoL versus selected factors before diagnosis, by the time of diagnosis or after diagnosis

To further explore factors positively or negatively associated with HRQoL, 34 variables collected from patients and medical records were selected and divided into three groups; *before diagnosis, by the time of diagnosis and after diagnosis.* Factors significantly associated with generic PedsQL4.0 or subscale scores are shown in Table 3.

**Table 3:** Selected factors present before, during or after diagnosis related to HRQoL as determined by PedsQL generic scale score and subscale scores

	*N*	Factors present							
		Overall Yes No HRQoL			Physical functioning	Emotional functioning	Social functioning	School functioning	Psychosocial functioning
	(Yes/No)	Mean (SD)	Mean (SD)	*Diff (CI)*	*Diff (CI)*	*Diff (CI)*	*Diff (CI)*	*Diff (CI)*	*Diff (CI)*
**Factors before diagnosis**									
School attendance before diagnosis < 50%	39/9	50 (18)	61 (21)	Ns	Ns	Ns	Ns	22 *(−39; −6)*	13 *(−26; −1)*
Using medications before diagnosis	17/30	45 (20)	56 (17)	Ns	Ns	Ns	13 *(−22; −3)*	Ns	10 *(−20; −1)*
**Factors during diagnostic evaluation**									
Occupational therapist engaged in diagnostic evaluation	31/18	53 (18)	49 (20)	Ns	Ns	−12 *(2–24)*	Ns	Ns	Ns
Physical therapist engaged in diagnostic evaluation	34/15	54 (17)	47 (22)	Ns	Ns	*−*14 *(3–26)*	Ns	Ns	Ns
Nutritionist engaged in diagnostic evaluation	5/44	61 (8)	51 (19)	Ns	Ns	−19 *(1–37)*	Ns	Ns	Ns
**Factors after diagnosis**									
Support from schoolteacher	42/7	55 (17)	41 (17)	−14 *(1–29)*	Ns	−19 *(4–35)*	Ns	Ns	−15 *(2–28)*
Been to physical therapy	27/22	50 (17)	57 (19)	Ns	Ns	Ns	Ns	14 (−27; −1)	Ns
Been to rehabilitation stay	14/35	43 (15)	57 (17)	14 *(−24; −3)*	Ns	23 *(−34; −11)*	14 *(− 22; −6)*	Ns	14 *(− 24; −5)*
Participation in leisure activity	21/41	59 (17)	46 (16)	−13 *(4–22)*	−18 *(6–31)*	Ns	− 13 *(5–21)*	−15 *(4–26)*	−10 *(2–19)*
Delayed school progression	37/12	49 (16)	65 (18)	16 *(− 27; −4)*	18 *(− 35; − 2)*	Ns	14 *(−23; − 6)*	21 *(− 35; −7)*	14 *(− 24; − 4)*
SMFQ Sum score 11 or higher (cut of, depression symptoms)	17/46	37 (12)	55 (16)	18 *(− 27; − 10)*	14 *(− 28; − 1)*	28 *(− 37; − 20)*	21 *(−28; − 14)*	13 *(−25; − 1)*	21 *(−29; − 14)*

Two-sided Independent Samples T-test. Overall HRQoL reported as mean (SD), and differences in overall HRQoL and subscale scores reported with 95% Confidence Interval or Ns if not statistically significant

Looking at the possible association between HRQoL and factors being present before the CFS/ME diagnose, we found that school attendance < 50% or using medications were associated with lower HRQoL in PedsQL subscales, but not with generic PedsQL4.0.

All adolescents had a physician involved in diagnostic evaluation. Beyond that, there were differences regarding the type of health personnel involved. Our analyses show that when either an occupational therapist, a physical therapist or a clinical nutritionist were involved, this was positively associated with the PedsQL4.0 subscale emotional functioning, but not with other subscales or generic score.

For the period after diagnosis, we found four factors associated with HRQoL. Support from schoolteacher was positively associated with generic PedsQL 4.0 (55 vs 41, CI (0.08–29)), and with the subscale scores for emotional and psychosocial functioning. Participation in leisure activity was positively associated with generic PedsQL4.0 (59 vs 46, CI (4–22)) and with the subscale scores for physical, social, school and psychosocial functioning. Been to rehabilitation stay was negatively associated with generic PedsQL4.0 (43 vs 57, CI (− 24; − 3)) and with subscale scores for emotional, social and psychosocial functioning. Been to physical therapy was negatively associated with school functioning. Delayed school progression was negatively associated with generic PedsQL4.0 (49 vs 65, CI (− 27; − 4)) and with subscale scores for physical, social, school and psychosocial functioning. We also found that possible clinically significant depression (SMFQ score equal to or greater than 11) was negatively associated with generic HRQoL (CI (− 27; − 10)) and with all dimensions. Findings from bivariate analyses along with clinical relevance were indicators for which factors to include in the multiple regression analysis.

### Multivariate analysis: HRQoL versus selected factors in a regression model

Multiple linear regression analysis was performed to predict HRQoL based on the four variables from after diagnosis, identified from bivariate analyses and with the most significant positive or negative association. The four variables were considered as clinically relevant and adequate to both health care, support from school and to the loss of important activities these adolescents suffer from*.* Dependent variable generic PedsQL 4.0 was normally distributed. Forthy-eight participants had responded to all predictor variables. Predictor variables correlated with HRQoL (Pearson’s *r* > .300 except for support from teacher r .290) (Table 4).

**Table 4:** Multiple Linear regression - predictors to HRQoL in adolescents diagnosed with CFS/ME

*N* = 48	Beta coefficient	(95%CI)	β	*p*
Constant	50	(34–66)		.000
Gender (a)	10	(−1–21)	.230	.079
Support from schoolteacher (b)(c)	10	(−3–23)	.200	.121
Delayed school progression (d)	−10	(−21–1)	−.249	.051
Participation in leisure activity (e)	8	(−2–18)	.211	.114
Been to rehabilitation stay (f)	−8	(− 18–2)	−.212	.104

**a)** female =0, male =1), **b)** support from schoolteacher no =0, yes =1, **c)** support from schoolteacher,

correlation coefficient to HRQoL .290 (<.3). **d)** school delay no = 0, yes =, 1, **e)** participate in

leisure activity no = 0, yes =1, **f)** been to rehabilitation stay no = 0, yes =1

*Model summary: Adj. R*^*2*^*.319, F Change 5.399, Sig (ANOVA) p = .001*

The results from the multiple regression analysis confirmed the associations from bivariate analyses. The regression model was significant at the level *p* = .001, explaining 32% of the variance. Support from schoolteacher and participation in leisure activities were positively associated with HRQoL, while negative associations came from delayed school progression, and having been to a rehabilitation stay. The regression coefficient for delayed school progression was − 10 (β − .249), and for support from schoolteacher 10 (β .200), indicating clinical relevance. We also looked at the multiple linear regression analysis without the four participants who reported recovery. The predictors were distributed similarly in the regression model, and with similar results.

Based on the difference in bivariate analyses between participants with and without depressive symptoms, we looked at the multiple regression analysis if excluding the group of 10 participants with depressive symptoms. With *n* = 38 participants the distribution of the predictors was still the same, but with regression coefficient for delayed school progression at − 14 (β − .378), and support from schoolteacher 10 (β .162). Participation in leisure activity, β .260, and been to rehabilitation stay, β − .143. Hence the model explained 23% of the variance (*p* = .019).

## Discussion

The primary aim of this study was to measure HRQoL in adolescents living with CFS/ME. Overall, HRQoL in this patient group was low. Secondary aims were to identify factors before, at time of and after diagnosis, associated with HRQoL. School absence higher than 50% before diagnostic evaluation, delayed school progression, having attended physical therapy or rehabilitation stay after diagnosis were associated with lower HRQoL. Occupational therapist, physical therapist or clinical nutritionist engaged in diagnostic evaluation were associated with higher HRQoL. After diagnosis, being supported by a schoolteacher, attending school or participating in leisure activities were associated with higher HRQoL. We found no correlation between duration of fatigue at time of enrolment and HRQoL, fatigue severity or depressive symptoms.

HRQoL in adolescents living with CFS/ME was low compared to healthy adolescents as reported by previous studies. Healthy adolescents typically scored 83 or higher, and adolescents with other chronic diseases scored from 66 to 77 [14–17]. Importantly, girls scored lower than boys in both generic and dimensional PedsQL4.0 and PedsQL-MFS. Similar results were earlier found in a Norwegian study from 2015 [16], and internationally [24]. The low HRQoL scores in CFS/ME adolescents suggest a need for new strategies to improve HRQoL.

School absence higher than 50% before diagnostic evaluation or delayed school progression were associated with lower HRQoL. Maintaining contact with school have in previous studies shown to be important [25, 26]. CFS/ME symptoms and the subsequent reduction in activities, socializing and school delay may lead to anxiety, depressive mood and increased tension [27]. Measures to maintain school progression to improve HRQoL for adolescents with CFS/ME should be considered.

Participation in physical therapy or a rehabilitation stay were associated with lower HRQoL. Rehabilitation programs with exercise, mobilization and body awareness typically delivered from physical therapists, are earlier described as effective in reducing medium- and long-term fatigue severity in CFS/ME patients [28]. These findings seemingly conflict with our findings, i.e. that physical therapy or rehabilitation stay were associated with lower HRQoL. The lack of knowledge and disagreement about strategies to improve HRQoL in these patients might contribute to disruption in therapeutic alliances with patients and parents, and to distrust in health care personnel [29–31]. Our findings might indicate that adolescents with low HRQoL are more likely to attend or being offered a rehabilitation stay, and that a long-term plan with regularly mapping of symptoms could be most helpful for health care personnel to plan an individualized rehabilitation stay.

On the other hand, occupational therapist, physical therapist and clinical nutritionist engaged in diagnostic evaluation were associated with higher HRQoL. Occupational and physical therapists are commonly engaged in adaption of management plans after CFS/ME diagnosis [1]. Our findings suggest engagement also in diagnostic evaluation. Engagement from nutritionists could potentially be important since approximately 10% of adolescents with CFS/ME suffer from eating-difficulties [13].

Importantly, we found that support from schoolteacher was associated with higher HRQoL. To meet responsive and caring teachers, get assistance from sympathetic counselors, and the possibility to have flexible schedules might be just as important as support and care from health care professionals [1]. The positive association between support from schoolteachers and emotional functioning may be related to prevention of depressive symptoms. The importance of meeting in small groups with peers, and cooperation between health care professionals and school is earlier described as helpful [27]. The ability of schoolteachers to support adolescents with CFS/ME who are not present at school might improve the cohesion with school society and secondary improve HRQoL.

School attendance could result in PEM from physical, cognitive and social activity. Socialization is recommended as a priority when adolescents with CFS/ME attend school, and adjustment of the curriculum is necessary [1]. Teaching via digital tools is a strategy that potentially benefit adolescents with CFS/ME.

Participation in leisure activities was associated with higher HRQoL. Perhaps this is because the healthiest adolescents are more likely to participate in leisure activities. However, participation in leisure activities should be further studied throughout the course of CFS/ME when searching for strategies to improve HRQoL. This may decrease stigmatization of adolescents with CFS/ME who participate in leisure activities, even if they don’t manage obligatory school activity in line with healthy adolescents.

We found no correlation between duration of fatigue at time of enrolment and HRQoL, fatigue severity or depressive symptoms. Adolescents with CFS/ME are not able to do the things they want, and they suffer from loss, disruption and coping barriers [1, 26, 27, 32]. A previous study found no statistical evidence between depressive symptoms and low HRQoL [16]. Rather the duration of fatigue before diagnosis, the demanding diagnostic process, lack of medical understanding and lack of positive prognosis information, might provoke anxiety since the adolescents perceive their CFS/ME as being permanent and threatening to their future hopes and dreams [6, 10]. Previous studies emphasize how important it is that professionals involved in the diagnostic evaluation and health care of adolescents after diagnosis of CFS/ME agree about treatment and communicate consistently [29]. According to Rowe [1] “Management of CFS/ME requires careful attention.”, and that the surroundings are aware and supportive in order to give the adolescents a potential to prevent depressive symptoms and gain hope of an active and productive future. To avoid increased symptoms and relapses, they need a long-term plan for health care with regularly mapping of symptoms, guidance on activity, and regularly adjustments to symptoms severity or improvement [1]. Collectively, our findings support this.

### Strengths and limitations

A strength to our study is that we explore the relationship between HRQoL and various factors especially during the period after diagnosis. Furthermore, our participants had a mean duration of fatigue close to 4 years which adds relevance to our findings. The diagnosis was verified according to the Jason criteria, and the diagnostic evaluation and health care after diagnosis at the two hospitals participating in this study are relatively uniform. A limitation to our study was a possibility to miss significant associations between HRQoL and factors before, by the time of or after diagnosis due to a small sample size. In the multiple regression analysis we only adjusted for gender, due to sample size. Bivariate analyzes were not corrected for multiple testing, and accordingly results should be cautiously interpreted. Sixty-three participants reported HRQoL with self-completed questionnaires, while only 48 responded to the telephone interview. This lost-to-follow-up bias resulted in exclusion of participants in the bivariate and multivariate analyses and could have been avoided by adding the questions from the telephone interview to the self-complete questionnaire. A further limitation was that patients with CFS/ME often have reduced cognitive function, and it may be difficult to remember exactly what occurred early in the disease course leading to recall-bias. Importantly, given our cross-sectional study design, it is impossible to conclude whether factors have a causal significance to HRQoL.

### Implications

**Fig. 2:**
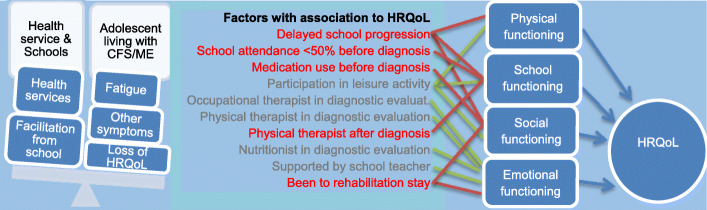
Factors found with association to HRQoL in adolescents living with CFS/ME

We still lack effective treatment of fatigue in CFS/ME patients, and despite effort from health-services and schools, HRQoL in adolescents with CFS/ME is low. A focus on strategies to improve psychosocial function, especially in relation to school, during diagnostic evaluation and after diagnosis might contribute to higher HRQoL (Fig. 2). Focus on participation in leisure activities in association to HRQoL is potentially needed to improve HRQoL and avoid stigmatization of CFS/ME adolescents. A long-term plan for health care with regularly mapping of symptoms from early stages of the disease might reveal data with importance to prevent the significant reduction of HRQoL, regarding both physical, social and emotional aspects [1]. Cooperation between schools, primary health care and hospitals when caring for adolescents with CFS/ME are of most importance.

For all patients with chronic health conditions, the goal of health care should be to restore them to the fullest health possible by improving symptom management, treatment adherence, and their ability to cope with their condition. For this reason, HRQoL may be just as important as biomedical measures when assessing patients with chronic health conditions like CFS/ME. The PedsQL4.0 is developed from the Worlds Health Organization’s definition of health and is a valid instrument for this purpose [33].

## Conclusion

In this cross-sectional study of adolescents with CFS/ME we found low HRQoL. The study identified new and possibly important factors associated with HRQoL. When exploring factors before, at the time of or after diagnosis associated with HRQoL, we found that school attendance, support from a schoolteacher and participation in leisure activities were associated with higher HRQoL. We also found associations to higher emotional functioning, when occupational therapist, physical therapist and clinical nutritionist were engaged in diagnostic evaluation. On the other hand, school absence higher than 50% before diagnostic evaluation, delayed school progression or having been to a rehabilitation stay were negatively associated with HRQoL. Early diagnosis, mapping of symptoms severity and HRQoL, maintaining school contact and early action to prevent depressive symptoms might be important to improve HRQoL in these patients. Limitations to our study design imply that future interventional studies are needed to confirm whether the identified factors can be used to improve HRQoL in adolescents with CFS/ME.

## Supplementary information

**Additional file 1: Supplemental Table 1.** HRQoL as measured by generic PedsQL4.0 versus selected factors before, at or after diagnosis **Supplemental Table 2.** Physical functioning versus selected factors before, at or after diagnosis. **Supplemental Table 3.** Emotional functioning versus selcted factors before, at or after diagnosis. **Supplemental Table 4.** Social functioning versus selected factors before, at or after diagnosis. **Supplemental Table 5.** School functioning versus selected factors before, at or after diagnosis. **Supplemental Table 6.** Psychosocial functioning versus selected factors before, at or after diagnosis.

## Data Availability

The dataset generated and analyzed during the current study is not public available due to ethical standards for treatment of patient data.

## Abbreviations

CFS/ME: Chronic fatigue syndrome/myalgic encephalomyelitis
PEM: Post exertional malaise
HRQoL: Health related quality of life
PedsQL4.0: Pediatric Quality of Life Inventory™ Generic Core scale version 4.0
PedsQL-MFS: Pediatric Quality of Life Inventory™ Multidimensional Fatigue scale
SMFQ: Short Mood and Feelings Questionnaire
DPHQ-N: De Paul Pediatric Health Questionnaire –Norwegian version
