# Supplementary material for: Health-related quality of life in Norwegian adolescents living with chronic fatigue syndrome
Source: Health Qual Life Outcomes. 2020 Jun 5;18:170. doi: 10.1186/s12955-020-01430-z (PMC7275299; doi:10.1186/s12955-020-01430-z)
Supplement: Supplementary file 1 — Additional file 1: Supplemental Table 1. HRQoL as measured by generic PedsQL4.0 versus selected factors before, at or after diagnosis Supplemental Table 2. Physical functioning versus selected factors before, at or after diagnosis. Supplemental Table 3. Emotional functioning versus selcted factors before, at or after diagnosis. Supplemental Table 4. Social functioning versus selected factors before, at or after diagnosis. Supplemental Table 5. School functioning versus selected factors before, at or after diagnosis. Supplemental Table 6. Psychosocial functioning versus selected factors before, at or after diagnosis. [file 12955_2020_1430_MOESM1_ESM.docx]

**Supplemental Table 1: HRQoL as measured by generic PedsQL4.0, variables from questionnaires and data collected from patient journals.**

|  |  |  |  |  |
| --- | --- | --- | --- | --- |
| **Generic PedsQL4.0** | **N**  **Yes/No** | **Mean** (SD)  **Yes** | **Mean** (SD)  **No** | *p* |
| **Factors before diagnosis**  Using medications before diagnosis  School attendance before diagnosis < 50 %  **Factors at diagnostic evaluation**  Diagnosed in outpatient clinic (not hospitalized)  Joint consultation by medical and psychiatric units  Child/adolescent psychiatry engaged in diagn. eval.  Nurse engaged in diagnostic evaluation  Nutritionist engaged in diagnostic evaluation  Physical therapist engaged in diagnostic evaluation  Occupational therapist engaged in diagn. evaluation  Educator engaged in diagnostic evaluation  Social worker engaged in diagnostic evaluation  Collaborative meeting with primary health care held  **Factors after diagnosis**  Individualized school schedule  Individualized examination plan  Home education by teacher  Organized schooldays  (with or without individualized school schedule)  Support from schoolteacher  Support from advisor in school  Been seeing pedagogic-psychologic services  Been seeing social worker  Been seeing nutritionist  Been seeing a psychiatric nurse or nurse  Been seeing a school nurse  Been to psycho motoric physical therapist  Been to physical therapist  Been seeing an occupational therapist  Been to child and adolescent psychiatry  Been seeing a general practitioner  Responsibility group  Been to rehabilitation stay  Participated in disease-specific coping courses  Received aids (i.e. wheelchair, taxi-drive to school)  Delayed school progression  Participate in leisure activity | 17/30  39/9  38/9  22/20  45/4  14/35  5/44  34/15  31/18  7/42  2/47  9/9  30/19  17/32  10/39  43/6  42/7  38/6  27/21  7/41  8/39  2/45  12/37  4/32  27/22  25/23  28/21  37/11  19/30  14/35  18/31  17/32  37/12  21/41 | 45.3 ± 19.8  49.7 ± 17.9  52.5 ± 16.9  51.2 ± 18.5  52.0 ± 18.8  48.6 ± 17.9  60.7 ± 8.1  53.7 ± 17.0  53.2 ± 17.6  63.3 ± 16.3  48.9 ± 20.0  48.3 ± 27.6  51.6 ± 16.3  49.5 ± 15.7  49.6 ± 22.6  52.0 ± 17.9  55.1 ± 17.4  52.2 ± 18.0  51.1 ± 18.5  47.5 ± 16.6  47.0 ± 12.7  58.7 ± 12.3  49.7 ± 14.4    60.9 ± 14.7  49.9 ± 16.6  51.5 ± 17.8  51.4 ± 17.8  52.0 ± 18.3  55.7 ± 19.4  43.1 ± 15.2  52.0 ± 18.6    49.3 ± 15.6  49.2 ± 16.3  59.4 ± 16.5 | 55.8 ± 17.4  61.0 ± 21.0  53.5 ± 25.1  53.0 ± 16.5  49.5 ± 19.0  53.0 ± 19.0  50.8 ± 19.2  47.4 ± 21.7  49.4 ± 20.4  49.9 ± 18.4  51.9 ± 18.7  58.1 ± 17.1  55.2 ± 20.3  54.9 ± 18.9  53.9 ± 16.7  60.3 ± 16.8  40.8 ± 16.7  47.6 ± 16.9  56.3 ± 16.9  53.0 ± 17.3  54.0 ± 19.0  51.8 ± 17.5  54.1 ± 18.9  52.3 ± 18.5  56.8 ± 18.9  53.0 ± 16.7  55.2 ± 18.1  57.1 ± 17.4  51.3 ± 16.9  57.0 ± 17.5  53.6 ±17.7  55.0 ± 18.9  64.7 ± 17.9  46.0 ± 16.5 | *.063*  *.104*  *.892*  *.730*  *.797*  *.457*  *.264*  *.273*  *.495*  *.076*  *.826*  *.378*  *.495*  *.327*  *.498*  *.289*  *.049*  *.566*  *.321*  *.439*  *.329*  *.586*  *.469*  *.381*  *.178*  *.759*  *.472*  *.416*  *.401*  *.012*  *.770*  *.292*  *.007*  *.004* |

**Supplemental Table 2: HRQoL as measured by PedsQL4.0– physical functioning dimension, variables from questionnaires and data collected from patient journals.**

| **PedsQL4.0 dimension- physical functioning** | **N**  **Yes/No** | **Mean** (SD)  **Yes** | **Mean** (SD)  **No** | *p* |
| --- | --- | --- | --- | --- |
| **Factors before diagnosis**  Using medications before diagnosis  School attendance before diagnosis < 50 %  Factors at diagnostic evaluation  Diagnosed in outpatient clinic (not hospitalized)  Joint consultation by medical and psychiatric units  Child/adolescent psychiatry engaged in diagn. eval.  Nurse engaged in diagnostic evaluation  Nutritionist engaged in diagnostic evaluation  Physical therapist engaged in diagnostic evaluation  Occupational therapist engaged in diagn. evaluation  Educator engaged in diagnostic evaluation  Social worker engaged in diagnostic evaluation  Collaborative meeting with primary health care held  **Factors after diagnosis**  Individualized school schedule  Individualized examination plan  Home education by teacher  Organized schooldays  (with or without individualized school schedule)  Support from schoolteacher  Support from advisor in school  Been seeing pedagogic-psychologic services  Been seeing social worker  Been seeing nutritionist  Been seeing a psychiatric nurse or nurse  Been seeing a school nurse  Been to psycho motoric physical therapist  Been to physical therapist  Been seeing an occupational therapist  Been to child and adolescent psychiatry  Been seeing a general practitioner  Responsibility group  Been to rehabilitation stay  Participated in disease-specific coping courses  Received aids (i.e. wheelchair, taxi-drive to school)  Delayed school progression  Participate in leisure activity | 17/30  39/9  38/9  22/20  45/4  14/35  5/44  34/15  31/18  7/42  2/47  9/9  30/19  17/32  10/39  43/6  42/7  38/6  27/21  7/41  8/39  2/45  12/37  4/32  27/22  25/23  28/21  37/11  19/30  14/35  18/31  17/32  12/37  41/21 | 36.8 ± 24.4  41.7 ± 25.7  44.3 ± 24.0  39.4 ± 26.3  43.2 ± 25.9  38.0 ± 24.1  45.9 ± 14.2  43.1 ± 26.3  42.7 ± 27.3  59.7 ± 21.4  25.0 ± 30.9  43.7 ± 37.0  44.1 ± 23.4  40.8 ± 25.0  40.5 ± 33.8  43.4 ± 25.3  46.6 ± 25.6  41.0 ± 24.5  42.2 ± 27.5  32.6 ± 27.9  35.2 ± 19.1  57.8 ± 2.2  38.3 ± 24.6  65.6 ± 23.2  39.7 ± 25.2  41.0 ± 26.4  43.9 ± 24.6  44.1 ± 27.1  52.3 ± 26.5  35.9 ± 16.5  45.2 ± 27.8  39.7 ± 26.9  40.2 ± 24.9  54.6 ± 24.8 | 47.6 ± 26.2  49.1 ± 28.8  42.4 ± 34.3  45.9 ± 25.6  38.3 ± 29.5  44.7 ± 26.7  42.4 ± 27.0  41.9 ± 25.9  42.8 ± 24.2  39.9 ± 25.8  43.5 ± 25.8  48.8 ± 27.7  45.6 ± 28.9  46.7 ± 25.8  45.7 ± 23.2  53.6 ± 27.1  33.2 ± 22.4  49.5 ± 23.7  48.5 ± 23.2  45.6 ± 23.9  46.0 ± 26.8  43.0 ± 25.3  46.7 ± 25.6  42.8 ± 25.4  50.8 ± 24.9  46.6 ± 22.9  45.7 ± 27.0  47.9 ± 20.4  39.9 ± 23.9    48,2 ± 27,6  44.4 ± 24.4  47.3 ± 24.6  58.5 ± 22.7  36.2 ± 22.2 | *.167*  *.447*  *.849*  *.420*  *.723*  *.417*  *.779*  *.876*  *.996*  *.061*  *.328*  *.744*  *.848*  *.444*  *.570*  *.361*  *.199*  *.433*  *.407*  *.202*  *.283*  *.001*  *.321*  *.097*  *.132*  *.441*  *.804*  *.665*  *.097*  *.064*  *.915*  *.324*  *.028*  *.004* |

**Supplemental Table 3: HRQoL as measured by PedsQL4.0 – emotional functioning dimension, variables from questionnaires and data collected from patient journals.**

| **PedsQL4.0 dimension-Emotional functioning** | **N**  **Yes/No** | **Mean** (SD)  **Yes** | **Mean** (SD)  **No** | *p* |
| --- | --- | --- | --- | --- |
| **Factors before diagnosis**  Using medications before diagnosis  School attendance before diagnosis < 50 %  **Factors at diagnostic evaluation**  Diagnosed in outpatient clinic (not hospitalized)  Joint consultation by medical and psychiatric units  Child/adolescent psychiatry engaged in diagn. eval.  Nurse engaged in diagnostic evaluation  Nutritionist engaged in diagnostic evaluation  Physical therapist engaged in diagnostic evaluation  Occupational therapist engaged in diagn. evaluation  Educator engaged in diagnostic evaluation  Social worker engaged in diagnostic evaluation  Collaborative meeting with primary health care held  **Factors after diagnosis**  Individualized school schedule  Individualized examination plan  Home education by teacher  Organized schooldays  (with or without individualized school schedule)  Support from schoolteacher  Support from advisor in school  Been seeing pedagogic-psychologic services  Been seeing social worker  Been seeing nutritionist  Been seeing a psychiatric nurse or nurse  Been seeing a school nurse  Been to psycho motoric physical therapist  Been to physical therapist  Been seeing an occupational therapist  Been to child and adolescent psychiatry  Been seeing a general practitioner  Responsibility group  Been to rehabilitation stay  Participated in disease-specific coping courses  Received aids (i.e. wheelchair, taxi-drive to school)  Delayed school progression  Participate in leisure activity | 17/30  39/9  38/9  22/20  45/4  14/35  5/44  34/15  31/18  7/42  2/47  9/9  30/19  17/32  10/39  43/6  42/7  38/6  27/21  7/41  8/39  2/45  12/37  4/32  27/22  25/23  28/21  37/11  19/30  14/35  18/31  17/32  37/12  21/41 | 51.2 ± 22.8  55.5 ± 18.2  57.2 ± 18.6  60.0 ± 21.4  57.8 ± 20.4  58.9 ± 22.0  74.0 ± 7.4  61.8 ± 16.7  61.9 ± 17.4  64.3 ± 16.7  77.5 ± 10.6  49.4 ± 26.6  58.0 ± 18.8  57.6 ± 17.5  59.0 ± 21.3  58.7 ± 20.4  61.5 ± 19.1  60.4 ± 21.2  60.0 ± 17.7  56.4 ±14.9  58.8 ± 19.4  55.0 ± 28.3  61.3 ± 17.9  46.3 ± 19.3  60.2 ± 20.1  61.2 ± 17.2  54.8 ± 21.8  56.9 ± 20.0  60.3 ± 20.2  42.9 ± 21.1  57.2 ± 18.4  57.4 ±14.6  56.9 ± 19.5  60.0 ± 17.2 | 59.5 ± 17.4  62.8 ± 25.8  60.0 ± 25.1  56.8 ± 15.1  51.3 ± 8.5  56.6 ± 19.0  55.3 ± 19.8  47.0 ± 22.5  49.2 ± 21.3  56.1 ± 20.1  56.4 ± 19.6  69.4 ± 11.8  60.0 ± 22.8  59.4 ± 21.8  58.7 ± 20.2  59.2 ± 20.6  42.1 ± 20.2  45.0 ± 15.8  58.8 ± 22.7  58.3 ± 20.6  58.7 ± 21.1  57.7 ± 19.6  58.0 ± 21.1  58.9 ± 19.8  57.0 ± 20.7  54.6 ± 22.0  64.1 ± 17.0  64.5 ± 21.7  57.8 ± 20.5  65.1 ± 16.2  59.7 ± 21.4  59.5 ± 22.8  64.6 ± 22.2  53.7 ± 20.6 | *.167*  *.324*  *.709*  *.576*  *.531*  *.709*  *.043*  *.014*  *.027*  *.312*  *.139*  *.056*  *.740*  *.779*  *.969*  *.960*  *.017*  *.097*  *.839*  *.820*  *.997*  *.854*  *.631*  *.235*  *.594*  *.249*  *.115*  *.280*  *.686*  *.000*  *.686*  *.724*  *.256*  *.232* |

**Supplemental Table 4: HRQoL as measured by PedsQL4.0– social functioning dimension, variables from questionnaires and data collected from patient journals.**

|  |  |  |  |  |
| --- | --- | --- | --- | --- |
| **PedsQL4.0 dimension- Social functioning** | **N**  **Yes/No** | **Mean** (SD)  **Yes** | **Mean** (SD)  **No** | ***p*** |
| **Factors before diagnosis**  Using medications before diagnosis  School attendance before diagnosis < 50 %  **Factors at diagnostic evaluation**  Diagnosed in outpatient clinic (not hospitalized)  Joint consultation by medical and psychiatric units  Child/adolescent psychiatry engaged in diagn. eval.  Nurse engaged in diagnostic evaluation  Nutritionist engaged in diagnostic evaluation  Physical therapist engaged in diagnostic evaluation  Occupational therapist engaged in diagn. evaluation  Educator engaged in diagnostic evaluation  Social worker engaged in diagnostic evaluation  Collaborative meeting with primary health care held  **Factors after diagnosis**  Individualized school schedule  Individualized examination plan  Home education by teacher  Organized schooldays  (with or without individualized school schedule)  Support from schoolteacher  Support from advisor in school  Been seeing pedagogic-psychologic services  Been seeing social worker  Been seeing nutritionist  Been seeing a psychiatric nurse or nurse  Been seeing a school nurse  Been to psycho motoric physical therapist  Been to physical therapist  Been seeing an occupational therapist  Been to child and adolescent psychiatry  Been seeing a general practitioner  Responsibility group  Been to rehabilitation stay  Participated in disease-specific coping courses  Received aids (i.e. wheelchair, taxi-drive to school)  Delayed school progression  Participate in leisure activity | 17/30  39/9  38/9  22/20  45/4  14/35  5/44  34/15  31/18  7/42  2/47  9/9  30/19  17/32  10/39  43/6  42/7  38/6  27/21  7/41  8/39  2/45  12/37  4/32  27/22  25/23  28/21  37/11  19/30  14/35  18/31  17/32  37/12  21/41 | 60.9 ± 18.7  66.7 ± 16.4  69.2 ± 15.9  68.9 ± 15.3  67.9 ± 16.1  66.4 ± 19.2  *72.0 ± 9.1  70.7 ± 12.8  70.3 ±13.2  75.0 ± 18.3  72.5 ± 10.6  63.3 ± 26.5  67.3 ± 14.2  67.1 ± 14.6  65.0 ± 17.5  68.7 ± 14.4  71.1 ± 12.9  70.0 ± 13.8  68.1 ± 15.5  73.6 ± 11.8  66.9 ± 11.3  72.5 ± 10.6  70.0 ± 8.3  73.8 ± 8.5  67.6 ± 12.4  68.4 ± 12.8  67.1 ± 14.9  68.9 ± 14.2  70.3 ± 17.1  59.3 ± 14.4  69.4 ± 16.1  67.1 ± 12.4  66.1 ± 12.7  75.2 ± 11.5 | 73.2 ± 13.2  77.8 ± 11.8  68.9 ± 17.6  69.5 ± 13.2  76.3 ± 14.9  69.4 ± 14.8  68.2 ± 16.6  63.7 ± 21.3  65.6 ± 20.1  67.5 ± 15.6  68.4 ± 16.3  74.4 ± 9.2  73.2 ± 13.5  70.9 ± 13.8  70.8 ± 13.1  75.8 ± 10.2  60.7 ± 18.6  59.2 ± 15.6  72.4 ± 11.6  68.2 ± 13.7  70.0 ± 15.0  68.7 ± 13.8  69.5 ± 15.6  69.8 ± 14.9  72.0 ± 15.8  69.6 ± 14.5  72.9 ± 12.5  72.7 ± 14.0  69.2 ± 12.0  73.7 ± 11.8  69.7 ± 13.0  70.9 ± 14.9  80.4 ± 12.9  62.4 ± 15.9 | *.012*  *.062*  *.958*  *.886*  *.322*  *.559*  *.618*  *.248*  *.320*  *.255*  .727  *.262*  *.160*  *.364*  *.251*  *.250*  *.071*  *.085*  *.301*  *.333*  *.580*  *.701*  .909  *.615*  *.275*  *.768*  *.161*  *.439*  *.793*  *.001*  *.956*  *.364*  *.001*  *.002* |

**Supplemental Table 5: HRQoL as measured by PedsQL4.0 – school functioning dimension, variables from questionnaires and data collected from patient journals.**

|  |  |  |  |  |
| --- | --- | --- | --- | --- |
| **PedsQL4.0 dimension – School functioning** | **N**  **Yes/No** | **Mean** (SD)  **Yes** | **Mean** (SD)  **No** | *p* |
| **Factors before diagnosis**  Using medications before diagnosis  School attendance before diagnosis < 50 %  **Factors at diagnostic point**  Diagnosed in outpatient clinic (not hospitalized)  Joint consultation by medical and psychiatric units  Child/adolescent psychiatry engaged in diagn. eval.  Nurse engaged in diagnostic evaluation  Nutritionist engaged in diagnostic evaluation  Physical therapist engaged in diagnostic evaluation  Occupational therapist engaged in diagn. evaluation  Educator engaged in diagnostic evaluation  Social worker engaged in diagnostic evaluation  Collaborative meeting with primary health care held  **Factors after diagnosis**  Individualized school schedule  Individualized examination plan  Home education by teacher  Organized schooldays  (with or without individualized school schedule)  Support from schoolteacher  Support from advisor in school  Been seeing pedagogic-psychologic services  Been seeing social worker  Been seeing nutritionist  Been seeing a psychiatric nurse or nurse  Been seeing a school nurse  Been to psycho motoric physical therapist  Been to physical therapist  Been seeing an occupational therapist  Been to child and adolescent psychiatry  Been seeing a general practitioner  Responsibility group  Been to rehabilitation stay  Participated in disease-specific coping courses  Received aids (i.e. wheelchair, taxi-drive to school)  Delayed school progression  Participate in leisure activity | 15/28  37/8  35/8  18/20  41/4  14/31  3/42  31/14  29/16  7/38  2/43  9/8  27/19  17/29  10/36  41/5  39/7  35/6  26/19  7/38  8/36  2/42  12/34  4/31  25/21  23/22  25/21  35/10  19/27  13/33  17/29  16/30  34/12  21/36 | 36.0 ± 26.0  39.3 ± 21.0  48.1 ± 26.6  41,9 ± 22.6  43.8 ± 23.0  37.5 ± 16.4  63.3 ± 12.6  44.7 ± 21.4  43.3 ± 21.3  56.4 ± 16.0  35.0 ± 21.2  39.4 ± 27.2  40.7 ± 19.2  37.6 ± 19.5  38.5 ± 26.0  41.8 ± 21.7  45.8 ± 22.5  43.6 ± 23.5  39.4 ± 21.6  36.4 ± 19.1  34.4 ± 13.5  50.0 ± 21.2  36.3 ± 12.6  55.0 ± 20.4  37.2 ± 18.7  41.1 ± 23.4  43.8 ± 19.7  42.6 ± 21.6  42.4 ± 21.9  38.5 ± 21.6  39.7 ± 23.1  37.8 ± 20.0  38.1 ± 20.5  50.5 ± 19.5 | 47.5 ± 20.7  61.9 ± 23.7  44.0 ± 21.8  44.0 ± 21.7  38.8 ± 25.3  46.0 ± 25.2  41.9 ± 23.0  40.4 ± 26.8  43.4 ± 26.4  40.9 ± 23.4  43.7 ± 23.2  43.8 ± 26.2  47.6 ± 26.1  47.1 ± 23.4  45.0 ± 21.3  58.0 ± 23.9  31.4 ± 17.5  35.8 ± 19.1  50.0 ± 22.7  44.0 ± 22.3  45.6 ± 24.0  42.6 ± 22.3  46.2 ± 24.4  43.1 ± 22.2  51.2 ± 24.2  44.6 ± 20.5  43.3 ± 25.5  47.5 ± 26.4  44.4 ± 22.9  45.6 ± 22.5  45.9 ± 21.8  46.7 ± 23.1  59.2 ± 20.4  35.7 ± 20.8 | *.121*  *.010*  *.645*  *.777*  *.681*  *.187*  *.120*  *.565*  *.982*  *.101*  *.605*  *.745*  *.307*  .169  *.421*  *.127*  *.118*  *.451*  *.119*  *.409*  *.213*  *.649*  *.082*  *.318*  *.032*  *.601*  *.945*  *.548*  *.759*  *.333*  *.372*  *.203*  *.004*  *.011* |

**Supplemental Table 6: HRQoL as measured by PedsQL4.0– psychosocial functioning dimension, variables from questionnaires and data collected from patient journals.**

|  |  |  |  |  |
| --- | --- | --- | --- | --- |
| **PedsQL4.0 dimension – Psychosocial functioning** | **N**  **Yes/No** | **Mean** (SD)  **Yes** | **Mean** (SD)  **No** | ***p*** |
| **Factors before diagnosis**  Using medications before diagnosis  School attendance before diagnosis < 50 %  **Factors at diagnostic evaluation**  Diagnosed in outpatient clinic (not hospitalized)  Joint consultation by medical and psychiatric units  Child/adolescent psychiatry engaged in diagn. eval.  Nurse engaged in diagnostic evaluation  Nutritionist engaged in diagnostic evaluation  Physical therapist engaged in diagnostic evaluation  Occupational therapist engaged in diagn. evaluation  Educator engaged in diagnostic evaluation  Social worker engaged in diagnostic evaluation  Collaborative meeting with primary health care held  **Factors after diagnosis**  Individualized school schedule  Individualized examination plan  Home education by teacher  Organized schooldays  (with or without individualized school schedule)  Support from schoolteacher  Support from advisor in school  Been seeing pedagogic-psychologic services  Been seeing social worker  Been seeing nutritionist  Been seeing a psychiatric nurse or nurse  Been seeing a school nurse  Been to psycho motoric physical therapist  Been to physical therapist  Been seeing an occupational therapist  Been to child and adolescent psychiatry  Been seeing a general practitioner  Responsibility group  Been to rehabilitation stay  Participated in disease-specific coping courses  Received aids (i.e. wheelchair, taxi-drive to school)  Delayed school progression  Participate in leisure activity | 17/30  39/9  38/9  22/20  45/4  14/35  5/44  34/15  31/18  7/42  2/47  9/9  30/19  17/32  10/39  43/6  42/7  38/6  27/21  7/41  8/39  2/45  12/37  4/32  27/22  25/23  28/21  37/11  19/30  14/35  18/31  17/32  37/12  21/41 | 50.2 ± 19.6  54,2 ± 15,9  57.3 ± 15.6  58.2 ± 16.3  57.1 ± 17.2  54.3 ± 16.2  70,3 ± 2,7  59,8 ± 14,1  59,1 ± 14,5  65.2 ± 15.0  61.7 ± 14.1  50.7 ± 24.5  56.0 ± 15.2  54.1 ± 13.8  54.2 ± 19.2  56.7 ± 16.4  59,8 ± 15,1  58.4 ± 16.4  56.0 ± 15.5  55.5 ± 11.2  53.3 ± 12.1  59.2 ± 20.0  55.8 ± 10.6  58.3 ± 11.8  55,5 ± 14,1  57.3 ± 14.9  55.9 ± 16.4  56.4 ± 15.6  57.6 ± 17.4  47.1 ± 16.8  56.0 ± 16.7  54.7 ± 12.2  54.3 ± 14.3  61,9 ± 13,2 | 60.5 ± 14.5  67,7 ± 17,7  59.7 ± 21.3  56.8 ± 14.0  55.4 ± 13.6  58.0 ± 17.1  55,4 ± 17,1  50,5 ± 20,8  53,2 ± 20,0  55.5 ± 16.8  56.7 ± 17.0  63.4 ± 13.2  60.3 ± 17.5  59.6 ± 17.0  58.6 ± 15.3  64.9 ± 12.4  44,8 ± 16,8  46.7 ± 15.2  61.0 ± 16.3  57.2 ± 16.2  58.5 ± 17.2  56.7 ± 15.6  58.3 ± 17.6  57.5 ± 17.1  60,3 ± 18,1  56.6 ± 16.3  60.1 ± 15.6  62.2 ± 18.4  57.7 ± 15.4  61,9 ± 13,9  58.7 ± 15.9  59.3 ± 17.7  68.1 ± 17.3  51,7 ± 16,6 | *.045*  *.029*  *.696*  *.756*  *.854*  *.493*  *059*  *.075*  *.247*  *.159*  *.688*  *.191*  *.374*  *.264*  *.445*  *.246*  *.020*  *.106*  *.285*  *.786*  *.425*  *.831*  *.653*  *.928*  *.298*  *.864*  *.369*  *.303*  *.990*  *.003*  *.578*  *.344*  *.008*  *.017* |
